# Prepregnancy Depression and Breastfeeding Duration: A Look at Maternal Age

**DOI:** 10.1155/2018/4825727

**Published:** 2018-11-01

**Authors:** Jordyn T. Wallenborn, Anny-Claude Joseph, Whitney C. Graves, Saba W. Masho

**Affiliations:** Virginia Commonwealth University, School of Medicine, Division of Epidemiology, Department of Family Medicine and Population Health, 830 East Main Street, Suite 821, P.O. Box 980212, Richmond, VA 23298-0212, USA

## Abstract

**Background:**

In the United States, major depressive disorder affects one in five women aged 20-40 years. During these childbearing years, depression can negatively impact maternal behaviors that are crucial for infant growth and development. This study examined the relationship between prepregnancy depression and breastfeeding duration by maternal age.

**Methods:**

Data from Phase 7 (2012-2013) of the Pregnancy Risk Assessment Monitoring System (N=62,483) were analyzed. Prepregnancy depression was dichotomized while breastfeeding duration was categorized as never breastfed, breastfed 8 weeks or less, and breastfed more than 8 weeks. Maternal age was a significant effect modifier; therefore, results were stratified by maternal age. Multinomial logistic regression was used to obtain odds ratios and 95% confidence intervals (CI).

**Results:**

For women aged 20-24, 25-29, and 30-34 years with prepregnancy depression, the odds of never breastfeeding and breastfeeding 8 weeks or less were significantly higher than in women with no history of prepregnancy depression. Notably, among women aged 25-29 with prepregnancy depression, the odds of never breastfeeding and breastfeeding 8 weeks or less were 93% (adjusted odds ratio (AOR) = 1.93, 95% CI =1.57-2.37) and 65% (AOR = 1.65, 95% CI = 1.37-1.99) higher compared to women with no history of prepregnancy depression, respectively.

**Conclusions:**

Having a history of poor mental health before pregnancy may increase the likelihood of premature breastfeeding cessation. A woman's mental health status before pregnancy should be considered in reproductive and prenatal care models. Efforts should be made to understand challenges women of specific age groups face when trying to breastfeed.

## 1. Introduction

The United States (US) has continuously exhibited discordance in breastfeeding initiation and duration rates. Approximately 83% of US infants initiated breastfeeding in 2013; however, only a quarter (24.9%) were exclusively breastfed through six months as recommended by clinicians [1, 2]. Although these current practices align with national targets (81.9% and 25.5%, respectively) [1], present disparities minimize the protective effects of breastfeeding on maternal and infant health outcomes. Estimates from 2014 show that suboptimal breastfeeding rates cost the US $3 billion in maternal and pediatric medical costs and $14 billion in premature death, a costly expenditure that could be offset by increasing the number of women who breastfeed in accordance with national recommendations [3]. Thus, exploring approaches to prevent early breastfeeding cessation is essential to reducing burdensome consequences for women, infants, and society.

Disparities in breastfeeding practices have been shown for numerous factors including maternal age. Reports show that younger women (less than 20 years of age) have decreased initiation or shorter durations of breastfeeding compared to their older counterparts [4–8]. In 2010, an estimated 6% of infants born to women less than 20 years old were exclusively breastfed at six months, nearly half of the rate for infants born to women 30 years or older (17.9%) [9]. This may be due to physical discomfort associated with lactation or an overall lack of intention to breastfeed resulting from plans to return to school or work, the stigma associated with breastfeeding in public, or the influence of a social support system that may not favor breastfeeding [7].

Research has also demonstrated that poor mental health is associated with breastfeeding duration [10, 11]. Studies have estimated 8-16% of women will have depression—with the majority of diagnoses occurring during childbearing years [12]. A previous longitudinal study displayed shorter breastfeeding durations among younger women and those with higher levels of prenatal and postpartum depressive symptoms [13]. Another cross-sectional study reported that women with postpartum depressive symptoms had a shorter exclusive and overall breastfeeding duration compared to women without depressive symptoms [14].

While studies have primarily focused on breastfeeding and maternal mental health during pregnancy or the postpartum period, few studies have assessed the impact of depression prior to pregnancy on breastfeeding. Evidence has shown that women with poor prepregnancy mental health conditions are more likely to experience mental health problems postpartum [15], a well-documented predictor of early breastfeeding cessation [13, 14]. Further, previous research has emphasized the importance of accounting for prepregnancy depression in practice efforts due to the increased risk of recurring mental health diagnoses across the prenatal period and life course thereafter [15].

Thus, examining the influence of prepregnancy depression on breastfeeding may help providers identify and appropriately intervene with women in age groups at highest risk of premature cessation. To assess these relationships, the current study aims to examine the association between prepregnancy depression and breastfeeding duration by maternal age.

## 2. Materials and Methods

The current study analyzed data from the 2012-2013 (Phase 7) Pregnancy Risk Assessment Monitoring System (PRAMS). PRAMS is a surveillance system funded by the Centers for Disease Control and Prevention (CDC) and state health departments [16]. Forty-seven states, New York City, Puerto Rico, the District of Colombia, and the Great Plains Tribal Chairmen's Health Board participate in PRAMS. Only California, Idaho, and Ohio do not participate. For all participating states, PRAMS collects information from women who had a recent live birth according to the state's birth certificate records. A combination of mailed questionnaires and a telephone survey are administered to mothers, who delivered within the past 2 to 4 months, to gather information on maternal and child health indicators. A detailed description of PRAMS methodology and questionnaires have been presented elsewhere [16].

This study included women with singleton births, no previous live births, whose infants were alive at the time of the interview and who had complete information on prepregnancy depression and breastfeeding duration—leaving 62,483 out of 72,540 women for analysis. This study limited the sample to women with no previous live birth to reduce variability and residual confounding associated with women's pre- and postpartum experiences. In accordance with previous literature [6, 17], breastfeeding duration (the main outcome) was categorized as never breastfed, breastfed 8 weeks or less, and breastfed more than 8 weeks with breastfeeding more than 8 weeks as the reference group. Because surveys were conducted at various time points (2-4 months postpartum), the 8-week cutoff was utilized to provide every women with an equal chance to breastfeed at the prespecified duration. Breastfeeding duration was based on the survey questions: “Did you ever breastfeed or pump milk to feed your new baby after delivery, even for a short period of time?”; “How many weeks or months did you breastfeed or pump milk to feed your baby?” The main exposure of interest was prepregnancy depression (yes; no). Participants were asked, “Before you got pregnant with your new baby, did a doctor, nurse, or other healthcare worker tell you that you had any of the following health conditions?” Depression before pregnancy was one of the listed conditions that participants could check as “yes” or “no”.

**Figure 1:**
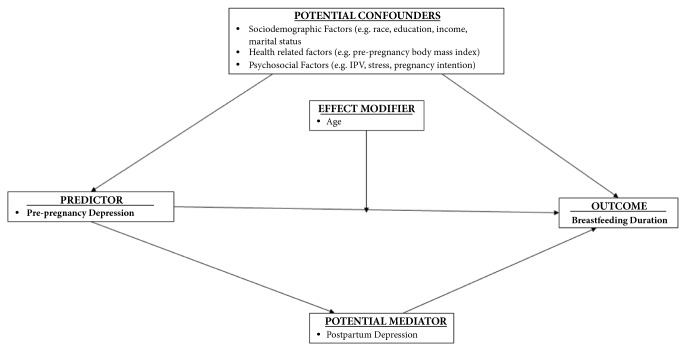
Directed acyclic graph of prepregnancy depression and breastfeeding duration.

Several maternal characteristics were identified in the literature as potential confounders (Figure 1). Sociodemographic factors included maternal age (<20; 20-24; 25-29; 30-34; ≥ 35), maternal race/ethnicity (White; Black; Hispanic; other), maternal education (less than high school; high school graduate; college or higher), marital status (married; not married), insurance used to pay for prenatal care (private; government; other; no coverage), and household income (< $20,000; $20,000-34,999; $35,000-$49,999; >$50,000). Health-related factors included prepregnancy body mass index (BMI) (underweight (<18.5 kg/m^2^); normal weight (18.5-24.9 kg/m^2^); overweight (25.0-29.9 kg/m^2^); obese (30.0+ kg/m^2^)). Intimate partner violence (no abuse, abuse before pregnancy, abuse during pregnancy, and abuse before and during pregnancy), total number of stressors (0; 1; 2; ≥3), and pregnancy intention (intended; not intended) were included as psychosocial factors.

All characteristics were summarized with weighted percentages and assessed using chi-square tests. All data presented are weighted to represent all women delivering live births among the included PRAMS states. Thus, the sample population reflects the population of women who gave birth within each state's general population. Logistic regression models were used to assess factors associated with prepregnancy depression using odds ratios (OR) and 95% confidence intervals (CI). The test for interaction between depression and age showed that age-related differences in the association were expected (*p* value <0.0001); thus, subsequent analyses were stratified by maternal age. A single multinomial logistic regression model was used to evaluate the relationship between prepregnancy depression and breastfeeding duration adjusting for all potential confounders that resulted in a 10% or greater change in crude estimates [18]. All analyses were conducted in SAS v9.4 (Cary, NC) with a significance level of 0.05.

## 3. Results

The majority (61.8%) of the study sample breastfed more than 8 weeks. Less than one-quarter (22.2%) of women breastfed 8 weeks or less, and 16.0% never breastfed. Approximately one in ten (10.9%) women reported experiencing prepregnancy depression. The majority of the study sample were non-Hispanic White (62.0%), had at least some college education (62.6%), and were married (61.6%). Chi-square analyses revealed a significant association between all potential confounding factors and prepregnancy depression (Table 1).

The unadjusted analysis showed that women with prepregnancy depression had higher odds of never breastfeeding (crude odds ratio (COR) = 1.87, 95% CI = 1.68-2.08) and breastfeeding 8 weeks or less (COR = 1.85, 95% CI = 1.68-2.03) compared to women who did not have prepregnancy depression. After adjusting for marital status, insurance used to pay for prenatal care, and total number of stressors, estimates remained significant though attenuated. Compared to women who did not have prepregnancy depression, women with prepregnancy depression had 59% (adjusted odds ratio (AOR) = 1.59, 95% CI = 1.42-1.77) and 50% (AOR = 1.50, 95% CI = 1.36-1.66) higher odds of never breastfeeding and breastfeeding 8 weeks or less, respectively (Table 2).

Table 3 displays results for the association between prepregnancy depression and breastfeeding duration stratified by maternal age. The final parsimonious model adjusted for marital status, insurance used to pay for prenatal care, and total number of stressors. Among women aged 35 years or older with prepregnancy depression, the odds of never breastfeeding and breastfeeding 8 weeks or less were 55% and 41% higher compared to women with no prepregnancy depression, respectively. Among women aged 30-34 years with prepregnancy depression, the odds of never breastfeeding and breastfeeding 8 weeks or less were 88% and 68% higher compared to women with no prepregnancy depression, respectively. Among women aged 25-29 years with prepregnancy depression, the odds of never breastfeeding and breastfeeding 8 weeks or less were 93% and 65% higher compared to women with no prepregnancy depression, respectively. Among women aged 20-24 years with prepregnancy depression, the odds of never breastfeeding and breastfeeding 8 weeks or less were 28% and 36% higher compared to women with no prepregnancy depression, respectively. No statistically significant relationship was found among women aged less than 20 years who had prepregnancy depression.

## 4. Discussion

We found that prepregnancy depression increases the likelihood of breastfeeding discontinuation in the postpartum period; however, this association varied by maternal age. To the authors' knowledge, there is only one prior study that assessed the relationship between prepregnancy mental health and breastfeeding practices among women in the US. A cross-sectional study using 2010-2011 PRAMS data assessed the association between having a prepregnancy mental health visit and breastfeeding initiation [19]. Women who reported a prepregnancy mental health visit for depression or anxiety were less likely to initiate breastfeeding compared to women who did not report a prepregnancy mental health visit [19]; however, due to limitations of earlier PRAMS phases, this study used a participant's prepregnancy mental health visit as a proxy of depression, rather than a reported mental health diagnosis by a healthcare provider. Moreover, this aforementioned study examined the influence of having a mental health history prior to pregnancy on breastfeeding initiation but did not explore the potential relationship between prepregnancy mental health status and breastfeeding duration or intensity in the postpartum period [19].

In our current study, prepregnancy depression increased the likelihood of a shorter breastfeeding duration. This may be the result of the strong relationship between prepregnancy depression and postpartum mental health. In fact, a previous study using data from a large health maintenance organization (HMO) found that roughly 56% of their study participants with a history of prepregnancy depression were diagnosed with prenatal depression in a subsequent pregnancy [20]. In addition, a cross-sectional study using the Medical Expenditure Panel Survey (MEPS) found that 52% of study participants who reported poor postpartum mental health had a previous history of poor mental health before or during pregnancy [15]. As a result, the recurrence of depression in the postpartum period may contribute to the decreased continuation of breastfeeding among impacted women, a breastfeeding barrier that has been well documented in previous literature [13, 14, 19, 21].

Findings from this study also indicated differing levels of association between prepregnancy depression and breastfeeding duration by age, with women aged 25 and older having a higher likelihood of breastfeeding noninitiation and early cessation. These results are inconsistent with other cross-sectional studies that demonstrated younger women are less likely to initiate and/or continue breastfeeding [5, 6]. Similarly, a previous review reported that younger maternal age is associated with breastfeeding discontinuation in the US and globally [22]. However, our study found no significant associations among women less than 20 years of age, while women with prepregnancy depression between the ages of 20 and 24 exhibited the lowest risk of noninitiation and early cessation among all age groups. These inconsistencies may be due to the focus on prepregnancy depression and breastfeeding duration in the current study, which was not examined in the previous literature [5, 6].

Moreover, the shorter breastfeeding duration exhibited among older, young adult women may be due to the average age of onset for most mood disorders, such as major depressive disorder, among women. Previous literature has reported that the average age of onset for mood disorders often ranges from 25 to 45 years of age [23]. This is an important consideration as women between the ages of 25 and 34 accounted for roughly 58% of the sample population in the current study and displayed the highest risks of early breastfeeding cessation. In addition, the average age of first-time mothers in the US has increased in recent years (2000: 24.9 years of age; 2014: 26.3 years of age) [24]. Consequently, older, young adult women may experience greater challenges coping with chronic depression in addition to embarking on motherhood. Therefore, it is possible that maternal age may have modified the severity of depression and subsequent breastfeeding practices among the sample population in the postpartum period. However, the intensity of depressive symptoms could not be assessed in the current study and is an important consideration for future research.

Causal inferences cannot be drawn from these findings due to the cross-sectional study design. All measures were self-reported and prone to recall and social desirability bias. Prepregnancy depression was not measured using a screening tool; therefore, this study could not capture women who had depression but were undiagnosed. Moreover, multiple confounders (i.e., insufficient milk supply, reports of physical breastfeeding challenges, maternal confidence, early return to work, and infant health status) could not be assessed as they were not available in the dataset. Lastly, this study did not assess exclusive breastfeeding as conducted in previous research [19, 25] due to the limited subsample of PRAMS states that collected this information and the focus on national trends for the current study.

## 5. Conclusions

Noted as a core indicator of preconception health [26], a woman's mental health status before pregnancy should be considered in reproductive and prenatal care models. Based on our findings, efforts should be made to increase breastfeeding promotion activities for women across all age groups. This is especially important for US women between the ages of 25 and 34 to help address the specific challenges they may face during pregnancy and the postpartum period. However, due to historical trends, additional research with larger samples of younger women (less than 20 years of age) may aid in exploring potential associations between prepregnancy mental health and reported breastfeeding durations that may be present among this group. This is an important consideration due to the high prevalence of pre- and postpregnancy depressive symptoms that have been reported among women of younger age [25, 27]. Future studies are also needed to explore how maternal age may modify the relationship between severity of a perinatal depression diagnosis and breastfeeding initiation and duration. Understanding barriers related to these factors may help improve optimal breastfeeding practices among women in the US.

## Figures and Tables

**Table 1:** Distribution of study population characteristics by prepregnancy depression (N=62,483).

	**Total**	**Pre-Pregnancy Depression**	
		**Yes**	**No**
	N (weighted %)	n (weighted row %)	n (weighted row %)
**Age** **∗** **∗** **∗**			
< 20	4979 (6.4)	653 (13.3)	4326 (86.7)
20-24	13981 (21.0)	1779 (11.6)	12202 (88.4)
25-29	17892 (30.1)	1881 (9.8)	16011 (90.2)
30-34	16466 (27.4)	1615 (8.6)	14851 (91.4)
≥ 35	9164 (15.2)	868 (8.7)	8296 (91.3)
**Race Ethnicity** **∗** **∗** **∗**			
Non-Hispanic White	31330 (62.0)	4016 (11.6)	27314 (88.4)
Non-Hispanic Black	10056 (13.0)	1029 (7.6)	9027 (92.4)
Non-Hispanic Other	10007 (9.4)	850 (7.0)	9157 (93.0)
Hispanic	10444 (15.6)	825 (6.9)	9619 (93.1)

**Education** **∗** **∗** **∗**			
Less than High School	9204 (13.5)	1277 (12.6)	7927 (87.5)
High School	15846 (23.9)	1973 (11.4)	13873 (88.6)
College or Higher	36576 (62.6)	3438 (8.8)	33138 (91.3)
**Marital Status** **∗** **∗** **∗**			
Married	36547 (61.9)	3067 (7.6)	33480 (92.4)
Not Married	25897 (38.1)	3719 (13.7)	22178 (86.3)
**Household Income** **∗** **∗** **∗**			
<$20,000	17585 (28.7)	2834 (15.0)	14751 (85.0)
$20,000-$34,999	11038 (19.7)	1216 (9.6)	9822 (90.4)
$35,000-$49,999	5399 (10.4)	517 (9.0)	4882 (91.0)
≥ $50,000	19439 (41.3)	1518 (7.6)	17921 (92.4)
**Insurance** **∗** **∗** **∗**			
Private	30606 (54.3)	2528 (7.9)	28078 (92.1)
Government	27693 (40.4)	3886 (12.7)	23807 (87.3)
No Coverage	1207 (2.2)	145 (12.0)	1062 (88.0)
Other	1948 (3.1)	136 (6.9)	1812 (93.1)

**WIC During Pregnancy** **∗** **∗** **∗**			
Yes	30312 (44.4)	4102 (12.6)	26210 (7.8)
No	31741 (55.6)	2637 (87.4)	29104 (92.2)
**Pre-Pregnancy BMI** **∗** **∗** **∗**			
Underweight (<18.5)	2771 (4.0)	334 (10.7)	2437 (89.3)
Normal (18.5-24.99)	29035 (50.1)	2612 (8.4)	26423 (91.6)
Overweight (25-29.99)	14465 (24.4)	1599 (9.7)	12866 (90.3)
Obese ( ≥ 30)	13595 (21.5)	2025 (14.2)	11570 (85.8)
**Intimate Partner Violence** **∗** **∗** **∗**			
No abuse	59525 (96.6)	6022 (9.3)	53503 (90.7)
Before pregnancy	805 (1.2)	218 (24.3)	587 (75.7)
During pregnancy	520 (0.7)	124 (21.3)	396 (78.7)
Both before and during pregnancy	1133 (1.5)	355 (28.9)	778 (71.1)

**Stressors** **∗** **∗** **∗**			
0	17163 (30.0)	826 (4.7)	16337 (95.3)
1	14414 (23.2)	1020 (6.6)	13394 (93.4)
2	10894 (17.2)	1110 (9.4)	9784 (90.6)
≥ 3	19763 (29.5)	3813 (18.1)	15950 (81.9)
**Pregnancy Intent** **∗** **∗** **∗**			
Intended	39915 (91.4)	3851 (8.9)	36064 (91.1)
Not intended	4313 (8.6)	714 (14.3)	3599 (85.7)
**Breastfeeding Duration** **∗** **∗** **∗**			
Never Breastfed	9479 (16.0)	1511 (13.5)	7968 (86.5)
≤ 8 weeks	14702 (22.2)	2072 (13.4)	12630 (86.6)
> 8 weeks	38302 (61.8)	3213 (7.7)	35089 (92.3)

Note. WIC refers to Special Supplemental Nutrition Program for Women, Infants, and Children.

BMI = body mass index. Sample size is unweighted; percentages are weighted.

*∗∗∗*Chi-square *p*<0.001.

**Table 2:** Association between prepregnancy depression and breastfeeding duration.

	**Never BF vs. Any BF >8 weeks**		**BF ≤ 8 weeks vs. Any BF >8 weeks**	
	**COR (95**%** CI)**	**AO** **R** ^**a**^ ** (95**%** CI)**	**COR (95**%** CI)**	**AO** **R** ^**a**^ ** (95**%** CI)**
Depression	1.87 (1.68-2.08)*∗∗∗*	1.59 (1.42-1.77)*∗∗∗*	1.85 (1.68-2.03)*∗∗∗*	1.50 (1.36-1.66)*∗∗∗*
No depression	1.00		1.00	

Note. BF = breastfeeding; COR = crude odds ratio; AOR = adjusted odds ratio; CI = confidence interval.

*∗∗∗p*<0.001.

^a^Parsimonious model controlling for marital status, insurance used to pay for prenatal care, and total number of stressors.

**Table 3:** Association between prepregnancy depression and breastfeeding duration stratified by age.

	**Never BF vs. Any BF >8 weeks**		**BF ≤ 8 weeks vs. Any BF >8 weeks**	
	**COR (95**%** CI)**	**AO** **R** ^**a**^ ** (95**%** CI)**	**COR (95% CI)**	**AO** **R** ^**a**^ ** (95**%** CI)**
*<20 years*				
Depression	0.97 (0.66-1.41)	1.12 (0.76-1.65)	1.22 (0.87-1.70)	1.28 (0.91-1.81)
No depression	1.00		1.00	
*20-24 years*				
Depression	1.31 (1.05-1.62)*∗*	1.28 (1.02-1.60)*∗*	1.54 (1.28-1.85)*∗∗∗*	1.36 (1.12-1.64)*∗∗*
No depression	1.00		1.00	
*25-29 years*				
Depression	2.22 (1.82-2.70)*∗∗∗*	1.93 (1.57-2.37) *∗∗∗*	1.99 (1.67-2.37)*∗∗∗*	1.65 (1.37-1.99) *∗∗∗*
No depression	1.00		1.00	
*30-34 years*				
Depression	2.28 (1.84-2.82)*∗∗∗*	1.88 (1.50-2.35) *∗∗∗*	1.92 (1.58-2.33)*∗∗∗*	1.68 (1.37-2.05) *∗∗∗*
No depression	1.00		1.00	
*≥35 years*				
Depression	1.90 (1.43-2.53)*∗∗∗*	1.55 (1.16-2.08)*∗∗*	1.65 (1.27-2.15)*∗∗*	1.41 (1.07-1.86)*∗*
No depression	1.00		1.00	

Note. BF = breastfeeding; COR = crude odds ratio; AOR = adjusted odds ratio; CI = confidence interval.

*∗p*<0.05; *∗∗p*<0.01; *∗∗∗p*<0.0001.

^a^Parsimonious model controlling for marital status, insurance used to pay for prenatal care, and total number of stressors.

## Data Availability

PRAMS is a publicly available, national, dataset.

## References

[1] Centers for Disease Control and Prevention (2016). *Breastfeeding report card, progressing toward national breastfeeding goals*.

[2] Gartner L. M., Morton J., Lawrence R. A. (2005). Breastfeeding and the use of human milk. *Pediatrics*.

[3] Bartick M. C., Schwarz E. B., Green B. D. (2017). Suboptimal breastfeeding in the United States: Maternal and pediatric health outcomes and costs. *Maternal & Child Nutrition*.

[4] Apostolakis-Kyrus K., Valentine C., Defranco E. (2013). Factors associated with breastfeeding initiation in adolescent mothers. *Journal of Pediatrics*.

[5] Bolton T. A., Chow T., Benton P. A., Olson B. H. (2009). Characteristics associated with longer breastfeeding duration: An analysis of a peer counseling support program. *Journal of Human Lactation*.

[6] Jones J. R., Kogan M. D., Singh G. K., Dee D. L., Grummer-Strawn L. M. (2011). Factors Associated With Exclusive Breastfeeding in the United States. *Pediatrics*.

[7] Sipsma H. L., Magriples U., Divney A., Gordon D., Gabzdyl E., Kershaw T. (2013). Breastfeeding behavior among adolescents: Initiation, duration, and exclusivity. *Journal of Adolescent Health*.

[8] Tucker C. M., Wilson E. K., Samandari G. (2011). Infant feeding experiences among teen mothers in North Carolina: Findings from a mixed-methods study. *International Breastfeeding Journal*.

[9] U.S. Department of Health and Human Services HRSA (2015). *Maternal and Child Health Bureau. Child Health USA 2014*.

[10] Hamdan A., Tamim H. (2012). The relationship between postpartum depression and breastfeeding. *International Journal of Psychiatry in Medicine*.

[11] Ystrom E. (2012). Breastfeeding cessation and symptoms of anxiety and depression: a longitudinal cohort study. *BMC Pregnancy and Childbirth*.

[12] Ko J. Y., Farr S. L., Dietz P. M., Robbins C. L. (2012). Depression and treatment among U.S. pregnant and nonpregnant women of reproductive age, 2005-2009. *Journal of Women's Health*.

[13] Mathews M. E., Leerkes E. M., Lovelady C. A., Labban J. D. (2014). Psychosocial predictors of primiparous breastfeeding initiation and duration. *Journal of Human Lactation*.

[14] Bascom E. M., Napolitano M. A. (2015). Breastfeeding Duration and Primary Reasons for Breastfeeding Cessation among Women with Postpartum Depressive Symptoms. *Journal of Human Lactation*.

[15] Witt W. P., Wisk L. E., Cheng E. R. (2011). Poor Prepregnancy and Antepartum Mental Health Predicts Postpartum Mental Health Problems among US Women: A Nationally Representative Population-Based Study. *Women's Health Issues*.

[16] Centers for Disease Contrl and Prevention. Pregnancy risk assessment monitoring system (PRAMS): Phase; 2010.

[17] Wallenborn J. T., Masho S. W. (2016). The Interrelationship between Repeat Cesarean Section, Smoking Status, and Breastfeeding Duration. *Breastfeeding Medicine*.

[18] Rothman K. J., Greenland S., Lash T. L. (2008). *Modern Epidemiology*.

[19] Wouk K., Stuebe A. M., Meltzer-Brody S. (2017). Postpartum Mental Health and Breastfeeding Practices: An Analysis Using the 2010–2011 Pregnancy Risk Assessment Monitoring System. *Maternal and Child Health Journal*.

[20] Dietz P. M., Williams S. B., Callaghan W. M., Bachman D. J., Whitlock E. P., Hornbrook M. C. (2007). Clinically identified maternal depression before, during, and after pregnancies ending in live births. *The American Journal of Psychiatry*.

[21] Pope C. J., Mazmanian D. (2016). Breastfeeding and postpartum depression: An overview and methodological recommendations for future research. *Depression Research and Treatment*.

[22] Whalen B., Cramton R. (2010). Overcoming barriers to breastfeeding continuation and exclusivity. *Current Opinion in Pediatrics*.

[23] Kessler R. C., Amminger G. P., Aguilar-Gaxiola S., Alonso J., Lee S., Üstün T. B. (2007). Age of onset of mental disorders: A review of recent literature. *Current Opinion in Psychiatry*.

[24] Mathews T. J., Hamilton B. E. (2016). Mean Age of Mothers is on the Rise: United States, 2000-2014. *NCHS Data Brief*.

[25] World Health Organization (1991). *Indicators for assessing breast-feeding practices: report of an informal meeting*.

[26] Robbins C., Boulet S. L., Morgan I. (2014). Core state preconception health indicators—pregnancy risk assessment monitoring system and behavioral risk factor surveillance system, 2009. *Morbidity and Mortality Weekly Report: Surveillance Summaries*.

[27] Katon W., Russo J., Gavin A. (2014). Predictors of Postpartum Depression. *Journal of Women's Health*.

